# Hyponatremia Is Associated with Worse Outcomes from Fall Injuries in the Elderly

**DOI:** 10.3390/ijerph14050460

**Published:** 2017-04-26

**Authors:** Spencer C. H. Kuo, Pao-Jen Kuo, Cheng-Shyuan Rau, Shao-Chun Wu, Shiun-Yuan Hsu, Ching-Hua Hsieh

**Affiliations:** 1 Division of Trauma Surgery, Department of Surgery, Kaohsiung Chang Gung Memorial Hospital and Chang Gung University College of Medicine, Niao-Song District, Kaohsiung City 833, Taiwan; spenc19900603@gmail.com (S.C.H.K.); ah.lucy@hotmail.com (S.-Y.H.); 2 Department of Plastic and Reconstructive Surgery, Kaohsiung Chang Gung Memorial Hospital and Chang Gung University College of Medicine, Kaohsiung City 833, Taiwan; bow110470@gmail.com; 3 Department of Neurosurgery, Kaohsiung Chang Gung Memorial Hospital and Chang Gung University College of Medicine, Kaohsiung City 833, Taiwan; ersh2127@cloud.cgmh.org.tw; 4 Department of Anesthesiology, Kaohsiung Chang Gung Memorial Hospital and Chang Gung University College of Medicine, Kaohsiung City 833, Taiwan; shaochunwu@gmail.com

**Keywords:** hyponatremia, fall injury, elderly, injury severity score, trauma, mortality

## Abstract

*Background*: Hyponatremia has been proposed as a contributor to falls in the elderly, which have become a major global issue with the aging of the population. This study aimed to assess the clinical presentation and outcomes of elderly patients with hyponatremia admitted due to fall injuries in a Level I trauma center. *Methods*: We retrospectively reviewed data obtained from the Trauma Registry System for trauma admissions from January 2009 through December 2014. Hyponatremia was defined as a serum sodium level <135 mEq/L, and only patients who had sustained a fall at ground level (<1 m) were included. We used Chi-square tests, Student *t*-tests, and Mann-Whitney U tests to compare elderly patients (age ≥65 years) with hyponatremia (n = 492) to those without (n = 2002), and to adult patients (age 20–64 years) with hyponatremia (n = 125). *Results*: Significantly more elderly patients with hyponatremia presented to the emergency department (ED) due to falls compared to elderly patients without hyponatremia (73.7% vs. 52.6%; OR: 2.5, 95% CI: 2.10–3.02; *p* < 0.001). Elderly patients with hyponatremia presented with a worse outcome, measured by significantly higher odds of intubation (OR: 2.4, 95% CI: 1.15–4.83; *p* = 0.025), a longer in-hospital length of stay (LOS) (11 days vs. 9 days; *p* < 0.001), higher proportion of intensive care unit (ICU) admission (20.9% vs. 16.2%; OR: 1.4, 95% CI: 1.07–1.76; *p* = 0.013), and higher mortality (OR: 2.5, 95% CI: 1.53–3.96; *p* < 0.001), regardless of adjustment by Injury Severity Scores (ISS) (AOR: 2.4, 95% CI: 1.42–4.21; *p* = 0.001). *Conclusions*: Our results show that hyponatremia is associated with worse outcome from fall-related injuries in the elderly, with an increased ISS, longer LOS, and a higher risk of death.

## 1. Background

The increasing proportion of elderly people in the population has become a major global issue in recent years; Taiwan is soon set to become an aged society [1,2]. In addition to old age-related medical problems, falls are particularly common and burdensome, causing physical injury and limiting social and physical ability, resulting in reduced independence and the potential for subsequent falls [3,4,5,6,7,8,9]. In emergency departments (ED), falls are often seen to cause trauma in the old-age population. Approximately one-third of the older population experiences at least one fall each year; a worldwide study of disease burden for 21 regions has estimated that falls are responsible for 35 million disability-adjusted life years [10,11]. However, vigilance among the elderly for preventing falls is still low, and the proportion of old aged community-dwellers at risk of falls who are aware of this risk and prioritize for preventive care is small [12].

Hyponatremia has been proposed to be among the factors related to elderly falls, along with neuromuscular diseases, hypoglycemia, postural hypotension, and medication use [13]. Hyponatremia is prevalent in the ED, and is more commonly seen in the elderly than in other adult populations [14]. The incidence of hyponatremia in the elderly is reported at almost 20%. In ambulatory care settings, it is also the most common electrolyte imbalance, occurring in approximately 7–11% of outpatient geriatric patients [14,15] and around 18% of long-term care residents [16]. Even mild hyponatremia has been shown to cause other forms of potential danger besides falls among the elderly, including altered mental status, osteoporosis and fractures, and gastrointestinal disturbances [13,16]. Furthermore, correction of hyponatremia in this population has the potential to impact socioeconomic status by reducing morbidity and mortality [17].

There is strong evidence that elderly trauma patients are at an increased risk of morbidity and mortality compared to younger patients [18,19,20]. In addition, because of the unique pathophysiology and physical capacity of the elderly with hyponatremia, their clinical presentation following a falling injury may differ from that of elderly patients without hyponatremia. However, there is limited information regarding their injury characteristics and outcome in such circumstances. Therefore, the purpose of this study was to assess the clinical presentation and outcomes of elderly patients with hyponatremia admitted for treatment following a falling injury in a Level I trauma center over a six-year period using trauma registry data.

## 2. Methods

We designed a retrospective study to review all patients whose data were entered into the Trauma Registry System between January 2009 and December 2014. Detailed patient information was retrieved from the Trauma Registry System of our institution, which included the following variables: age, gender, co-morbidities such as cerebral vascular accident (CVA), hypertension (HTN), coronary artery disease (CAD), congestive heart failure (CHF), diabetes mellitus (DM), end-stage renal disease (ESRD), and dementia, as well as vital signs, injury severity score (ISS), and serum sodium level on arrival; Glasgow coma scale (GCS) score; abbreviated injury scale (AIS); hospital length of stay (LOS); intensive care unit (ICU) LOS; in-hospital mortality; procedures performed in the ED; and associated trauma in each body region. This study was approved by the institutional review board (IRB) of the Kaohsiung Chang Gung Memorial Hospital (IRB approved No. 104-8790B), a 2400-bed facility and Level I regional trauma center that provides care to trauma patients primarily from Southern Taiwan. 

In this study, hyponatremia and normonatremia are defined as serum sodium levels <135 mEq/L and between 135 and 145 mEq/L, respectively. Only the patients having sustained a fall <1 m, indicating a ground level fall, were included. Elderly patients were defined as patients aged ≥65 years, while adult patients were defined as patients aged 20–64 years. Patients with hypernatremia were excluded from the study. To evaluate the clinical presentation of the elderly with hyponatremia and having sustained a falling injury, elderly patients with hyponatremia (n = 492) were compared with elderly patients without hyponatremia (n = 2002), and with adult patients with hyponatremia (n = 125) using the SPSS v.20 statistical software (IBM, Armonk, NY, USA). For categorical variables, Chi-square tests were used to determine the significance of the associations between the predictor and outcome variables. For continuous variables, student *t*-tests were applied to analyze normally distributed data, while Mann-Whitney U tests were used to compare non-normally distributed data. Univariate logistic regression analyses were initially performed to identify the significant predictor variables of the injury or mortality risk. The corresponding crude odds ratios (ORs) with 95% confidence intervals (CIs) for each variable were obtained. We also estimated the adjusted odds ratio (AOR) and 95% CI for mortality through stepwise model selection of a multiple regression model that was adjusted by controlling the ISS or with the additional confounding variable of underlying co-morbidities (CVA, HTN, CAD, CHF, DM, ESRD, and dementia). All of the results were presented as the mean ± standard deviation. A *p*-value < 0.05 was considered statistically significant.

This study was pre-approved by the Institutional Review Board (IRB) of the Chang Gung Memorial Hospital (approval number No. 104-8790B). Informed consent was waived according to IRB regulations.

## 3. Results

**Figure 1:**
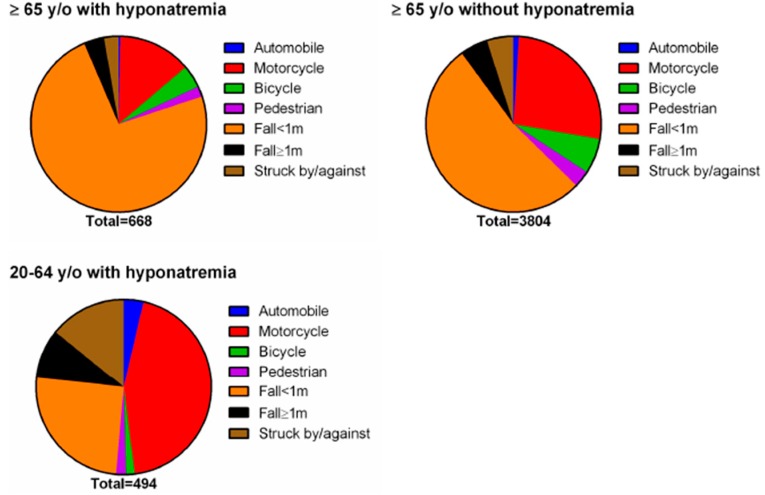
The relative proportions of different trauma mechanisms among elderly and adult patients with or without hyponatremia in the emergency department. y/o = years old.

As shown in Figure 1, for elderly patients with hyponatremia, 492 out of 668 patients (73.6%) were injured due to a fall. Other injuries included motorcycle accidents (90/668; 13.5%), bicycle accidents (28/668; 4.2%), falls from height ≥1 m (25/668; 3.7%), being struck by/against objects (18/668; 2.7%), pedestrians in an accident (13/668; 1.9%), and automobile accidents (2/668; 0.3%). For elderly patients without hyponatremia, the leading causes of injury were falls (2002/3804; 52.6%), followed by motorcycle (1017/3804; 26.7%) and bicycle (246/3804; 6.5%) accidents, and the leading causes of injury for adult patients with hyponatremia were motorcycle accidents (219/494; 44.3%), falls (125/494; 25.3%), and being struck by/against objects (70/494; 14.2%).

Significantly more elderly patients with hyponatremia presented to the ED due to falls compared with elderly patients without hyponatremia (73.7% vs. 52.6%; OR: 2.5, 95% CI: 2.10–3.02; *p* < 0.001). The mean serum sodium levels were 130.5 ± 4.1 mEq/L, 138.8 ± 2.3 mEq/L, and 130.7 ± 4.6 mEq/L in elderly patients with hyponatremia, elderly patients without hyponatremia, and adult patients with hyponatremia, respectively (Table 1). Significantly less elderly patients with hyponatremia had hemoglobin levels of ≥10.0 when compared with elderly patients without hyponatremia, while more elderly with hyponatremia patients are anemic and had hemoglobin levels between 8.0–9.9 and of ≤7.9 in comparison with elderly patients without hyponatremia. Elderly patients with hyponatremia had higher odds of CVA, CAD, DM, and ESRD than elderly patients without hyponatremia, and higher odds of CVA, HTN, CAD, ESRD, and dementia than adult patients with hyponatremia. There was no significant difference in GCS scores between the elderly with and without hyponatremia or between the elderly and adult patients with hyponatremia. A significantly higher ISS was found in the elderly patients with hyponatremia than in those without (9.8 ± 4.3 vs. 9.2 ± 4.2, respectively; *p* = 0.008); however, their ISS was not significantly higher than that of adult patients with hyponatraemia. Analysis of AIS revealed that the elderly patients with hyponatremia had sustained significantly higher rates of thoracic injury than elderly patients without hyponatremia (4.5% vs. 2.5%, respectively; *p* = 0.026) but lower rates of injury to the extremities (75.8% vs. 80.2%, respectively; *p* = 0.035). 

In addition, compared to adult patients with hyponatremia, the elderly patients with hyponatremia had sustained significantly lower rates of head/neck injury (23.2% vs. 32.8%, respectively; *p* = 0.029). Elderly patients with hyponatremia had higher odds of mortality than elderly patients without hyponatremia (OR: 2.5, 95% CI: 1.53–3.96; *p* < 0.001). Even when mortality was adjusted using ISS scores or ISS scores with the co-morbidities, higher mortality rate (AOR: 2.4, 95% CI: 1.42–4.21; *p* = 0.001 and AOR: 2.5, 95% CI: 1.41–4.32; *p* = 0.001, respectively) was noted in elderly patients with hyponatremia than in those without. No significant difference was found between the mortality rates of elderly and adult patients with hyponatremia, even after adjustment for the ISS score and other co-morbidities. Furthermore, the hospital LOS of elderly patients with hyponatremia was significantly longer than that of elderly patients without hyponatremia (average 11 days vs. 9 days; *p* < 0.001). A higher proportion of elderly patients with hyponatremia were admitted to the ICU than elderly patients without hyponatremia (20.9% vs. 16.2%; OR: 1.4, 95% CI: 1.07–1.76; *p* = 0.013), while the ICU LOS was not significantly different between the two groups. Compared to adult patients with hyponatremia, elderly patients with hyponatremia did not show significantly longer hospital or ICU LOS, nor was there a difference in the proportion of patients admitted to the ICU.

Upon arrival at the ED, no significant differences were found for GCS of <13, systolic blood pressure (SBP) of <90 mmHg, or heart rate of >100 beats/min between the elderly patients with or without hyponatremia (Table 2). More elderly patients with hyponatremia were intubated compared with elderly patients without hyponatremia (2.4% vs. 1.0%; OR: 2.4, 95% CI: 1.15–4.83; *p* = 0.025). Furthermore, no significant differences were found in the odds of needing chest tube insertion or blood transfusions at the ED between the two groups of elderly patients. In comparison with adult patients with hyponatremia, the elderly patients with hyponatremia had significant lower odds of SBP <90 mmHg (1.8% vs. 27.2%; OR: 0.1, 95% CI: 0.02–0.11; *p* < 0.001) but higher odds of heart rates >100 beats/min (17.5% vs. 1.6%; OR: 13.0, 95% CI: 3.16–53.70; *p* < 0.001). Neither group had significantly higher odds for requiring intubation, chest tube insertion, or blood transfusions at the ED.

Those injuries with significant difference in the affected body regions between groups are shown in Table 3, while information on all injuries is shown in the Supplementary Materials Table S1. Elderly patients with hyponatremia sustained a significant higher incidence of thoracic vertebral fracture, but fewer radial and ulnar fractures than elderly patients without hyponatremia. In contrast, compared to those adult patients with hyponatremia, elderly patients with hyponatremia sustained a significantly lower percentage of cranial fractures, epidural hematoma, and intracerebral hematoma, but a higher percentage of femoral fracture.

## 4. Discussion

As one of the most common electrolyte imbalance disorders among the elderly, hyponatremia was previously reported as a predictor of falls in a geriatric trauma population [13]. In this study, approximately 2.5-fold more elderly patients with hyponatremia presented to the ED due to falls than elderly patients without hyponatremia, suggesting a correlation between hyponatremia and elderly falls, consistent with similar reports in the literature. Asymptomatic hyponatremia has been reported to significantly increase the OR of falls in a population of ED admissions when controlling for multiple covariates (AOR: 67.43, 95% CI: 7.48–607.42; *p* < 0.001), which is believed to be a result of the significant gait and attention impairments that come with hyponatremia [21]. In a population of community-dwelling individuals, Gunathilake et al. demonstrated approximately 8-fold higher odds of falls in the elderly with hyponatremia after adjusting for age, sex, and diuretic use [22]. The study also reported that a serum sodium drop of 5 mEq/L (from 135 to 130 mEq/L) was correlated with a 32% increased risk of fall (OR: 1.32, 95% CI: 1.04–1.64) [22]. Tolouian et al. also reported increased odds of falls when adjusting for age (AOR: 4.80, 95% CI: 1.06–21.67; *p* = 0.04) in a population of patients admitted to the hospital for hip fractures secondary to a fall [17].

In this study, elderly patients with hyponatremia had a higher prevalence of CVA, HTN, CAD, and ESRD than adult patients with hyponatremia, which may be due to the age differences between the two groups. Furthermore, co-morbidities such as CVA, CAD, and DM were significantly more common in elderly patients with hyponatremia than in elderly patients without hyponatremia. In the literature, the formation of coronary atherosclerotic plaques in CAD was reported to be associated with serum sodium concentration. A multiple linear regression analysis demonstrated that sodium level was significantly and independently associated with the Gensini score [23]. This measure defines the severity of coronary atherosclerosis and is computed by assigning a severity score to each coronary stenosis according to the degree of luminal narrowing and its geographic importance. Further multinomial logistic regression analysis concluded that hyponatremia is a risk factor for higher Gensini score, indicating greater severity of atherosclerotic plaque formation [24]. In addition, the essential pathophysiology of DM is also closely related to electrolyte regulation, especially sodium regulation, and DM serves as a well-known cause of dysnatremias through *various* underlying mechanisms [25,26]. A previous study by Beukhof et al. reported that DM is associated with hyponatremia independent of drugs or hyperglycemia [27]. In community subjects aged ≥55 years, DM has also been reportedly associated with hyponatremia (OR = 1.98, 95% CI: 1.47–2.68) [26]. Proposed underlying mechanisms for DM and hyponatremia include altered vasopressin metabolism and the interaction between insulin and vasopressin—both of which function in the renal collecting duct—as well as the reabsorption of more hypotonic fluid due to slower stomach emptying [28,29,30]. Strict control of serum glucose was reported to be beneficial to prevent dysnatremias in these diabetic patients [31].

According to previous studies, patients with hyponatremia have higher mortality rates and longer hospital LOS [32,33,34,35]. With a cohort of 4123 elderly patients, Terzian et al. proposed that after adjusting for age, sex, LOS, and several clinical factors, hyponatremia was a significant independent predictor of mortality [32]. In a study of patients admitted with CHF, hyponatremia was independently associated with major complications during hospitalization, longer LOS, and greater inpatient mortality [33]. Significantly greater in-hospital and 60-day mortality was documented in patients with hyponatremia who were hospitalized for worsening heart failure compared with patients with normal or high serum sodium [34]. In a study conducted on patients with acute ST-elevation myocardial infarction, hyponatremia at admission or during the first 72 h of hospitalization was an independent predictor of 30-day mortality [35]. In the present study, elderly patients with hyponatremia had approximately 2.5-fold higher odds of mortality than elderly patients without hyponatremia, regardless of adjustment by other co-morbidities and ISS scores. In contrast, the mortality rates of elderly and adult patients with hyponatremia were not significantly different, even after adjustment with other co-morbidities and ISS scores. This phenomenon implies that hyponatraemia, but not age, has an important impact on increased mortality of trauma patients. Furthermore, elderly patients with hyponatremia had worse outcomes than elderly patients without hyponatremia, since they had significantly higher odds of intubation at ED, a longer in-hospital LOS, and a higher proportion of ICU admission. 

Hyponatremia has also been reported to be a risk factor for elderly people with osteoporosis and fragility fractures [36,37]. Cumming et al. claimed that in elderly patients with fragility fractures, hyponatremia was highly prevalent, with the most common potentially causative factors including dehydration, along with the prescription of thiazide diuretics and proton pump inhibitors [38]. Gankam Kengne et al. proposed that even mild asymptomatic hyponatremia is associated with bone fractures among the ambulatory elderly (AOR: 4.16, 95% CI: 2.24–7.71) [39]. Sandhu et al. reported that the incidence of hyponatremia in patients with fractures was more than twice that of the non-fracture patients (9.1% and 4.1%, respectively; *p* = 0.007), with the degree of hyponatremia being mild to moderate (mean serum sodium level = 131 ± 2 mEq/L) [40]. In this study, elderly patients with hyponatremia had higher odds of thoracic vertebral fracture, but a lower rate of radial and ulnar fracture, than elderly patients without hyponatremia. Some results from this study are contrary those of previous reports; however, because the elderly patients with hyponatremia had a significantly higher ISS, and the impact mechanisms of the falls were unknown, the reasons behind the lower radial and ulnar fracture rates remain to be answered.

There are several limitations to this study. The retrospective design has an inherent selection bias, which is a major limitation here. A second source of potential bias may come from the exclusion of patients declared dead (either on hospital arrival or at the accident scene) and injured patients who were discharged against the advice of the ED. In addition, patient medication use, muscle power, sugar level, and other electrolyte imbalances were not recorded in the trauma registry system and could not be evaluated in this study, but represent other important risk factors for falls. We also lacked data regarding the circumstances of the injury. Furthermore, the documented indications for ICU discharge may result in a bias in the evaluation of the ICU LOS. Finally, the study population was limited to a single urban trauma center in southern Taiwan, which may not be representative of other populations. Moreover, a further subgroup analysis according to the sodium level of these hyponatremia may provide more information but was not performed because of expected reduced statistical power by decreased sample size.

## 5. Conclusions

This study based on trauma admissions at a Level I trauma center shows that elderly patients with hyponatremia that presented to the ED from January 2009 to December 2014 were more likely to have experienced injuries from falls than those without hyponatremia. These patients also had anemia and co-morbidities (CVA, CAD, and DM), a higher ISS, a longer hospital LOS, higher ICU admission rates, and higher mortality rates.

## Figures and Tables

**Table 1:** Demographics and injury characteristics of elderly patients with hyponatremia who had sustained falls.

Variables (Fall < 1 m)	Patients ≥ 65 y/o with Hyponatremia n = 492		Patients ≥ 65 y/o without Hyponatremia n = 2002		Patients 20–64 y/o with Hyponatremia n = 125		OR (95% CI) *p* Patients ≥ 65 y/o with Hyponatremia vs. Patients ≥ 65 y/o without Hyponatremia			OR (95% CI) *p* Patients ≥ 65 y/o with Hyponatremia vs. Patients 20–64 y/o with Hyponatremia		
Na Level	112~134		135~147		107~134		-		-	-		-
Mean ± SD	130.5	±4.1	138.8	±2.3	130.7	±4.6	-		-	-		-
Gender												
Male	181	(36.8)	584	(29.2)	76	(60.8)	**1.4**	**(1.15–1.74)**	**0.001**	**0.4**	**(0.25–0.56)**	**<0.001**
Female	311	(63.2)	1418	(70.8)	49	(39.2)	**0.7**	**(0.58–0.87)**	**0.001**	**2.7**	**(1.78–3.99)**	**<0.001**
Hemoglobin	n = 492		n = 2001		n = 125							
≥10.0	367	(74.6)	1681	(84.0)	98	(78.4)	**0.6**	**(0.44–0.71)**	**<0.001**	0.8	(0.51–1.30)	0.417
8.0–9.9	90	(18.3)	272	(13.6)	18	(14.4)	**1.4**	**(1.10–1.85)**	**0.008**	1.3	(0.77–2.31)	0.357
≤7.9	35	(7.1)	48	(2.4)	9	(7.2)	**3.1**	**(1.99–4.87)**	**<0.001**	1.0	(0.46–2.11)	1.000
Co-Morbidity												
CVA	88	(17.9)	275	(13.7)	9	(7.2)	**1.4**	**(1.05–1.78)**	**0.022**	**2.8**	**(1.37–5.75)**	**0.002**
HTN	327	(66.5)	1269	(63.4)	56	(44.8)	1.1	(0.93–1.41)	0.209	**2.4**	**(1.64–3.64)**	**<0.001**
CAD	74	(15.0)	183	(9.1)	8	(6.4)	**1.8**	**(1.32–2.35)**	**<0.001**	**2.6**	**(1.21–5.52)**	**0.011**
CHF	17	(3.5)	54	(2.7)	4	(3.2)	1.3	(0.74–2.25)	0.222	1.1	(0.36–3.28)	1.000
DM	201	(40.9)	606	(30.3)	59	(47.2)	**1.6**	**(1.30–1.95)**	**<0.001**	0.8	(0.52–1.15)	0.224
ESRD	37	(7.5)	89	(4.4)	25	(20.0)	**1.7**	**(1.18–2.60)**	**0.006**	**0.3**	**(0.19–0.57)**	**<0.001**
Dementia	33	(6.7)	112	(5.6)	1	(0.8)	1.2	(0.81–1.81)	0.389	8.9	(1.21–65.83)	0.007
GCS	14.5	±1.8	14.5	±1.7	14.0	±2.6	-		0.415	-		0.096
1–8	14	(2.8)	48	(2.4)	8	(6.4)	1.2	(0.65–2.18)	0.627	0.4	(0.18–1.05)	0.063
9–12	23	(4.7)	73	(3.6)	7	(5.6)	1.3	(0.80–2.09)	0.295	0.8	(0.35–1.97)	0.816
13–15	455	(92.5)	1881	(94.0)	110	(88.0)	0.8	(0.54–1.16)	0.255	1.7	(0.89–3.17)	0.147
ISS	9.8	±4.3	9.2	±4.2	9.6	±5.8	-		**0.008**	-		0.809
1–8	67	(13.6)	394	(19.7)	39	(31.2)	**0.6**	**(0.49–0.85)**	**0.002**	**0.3**	**(0.22–0.55)**	**<0.001**
9–15	358	(72.8)	1369	(68.4)	56	(44.8)	1.2	(0.99–1.54)	0.064	**3.3**	**(2.20–4.93)**	**<0.001**
16–24	49	(10.0)	194	(9.7)	24	(19.2)	1.0	(0.74–1.43)	0.865	**0.5**	**(0.27–0.79)**	**0.006**
≥25	18	(3.7)	45	(2.2)	6	(4.8)	1.7	(0.95–2.88)	0.079	0.8	(0.29–1.94)	0.603
AIS												
Head/Neck	114	(23.2)	384	(19.2)	41	(32.8)	1.3	(1.00–1.61)	0.051	**0.6**	**(0.40–0.95)**	**0.029**
Face	12	(2.4)	74	(3.7)	5	(4.0)	0.7	(0.35–1.21)	0.214	0.6	(0.21–1.74)	0.358
Thorax	22	(4.5)	51	(2.5)	7	(5.6)	**1.8**	**(1.08–2.98)**	**0.026**	0.8	(0.33–1.89)	0.636
Abdomen	9	(1.8)	53	(2.6)	2	(1.6)	0.7	(0.34–1.40)	0.336	1.1	(0.24–5.37)	1.000
Extremity	373	(75.8)	1605	(80.2)	84	(67.2)	**0.8**	**(0.61–0.98)**	**0.035**	1.5	(1.00–2.34)	0.053
Mortality	28	(5.7)	48	(2.4)	7	(5.6)	**2.5**	**(1.53–3.96)**	**<0.001**	1.0	(0.43–2.39)	1.000
ISS(AOR)	-		-		-		**2.4**	**(1.42–4.21)**	**0.001**	1.4	(0.53–3.90)	0.473
ISS+CO(AOR)	-		-		-		**2.5**	**(1.41–4.32)**	**0.001**	2.7	(0.49–4.49)	0.255
Hospital LOS (days)	11.1	±1.0	9.1	±8.0	11.6	±11.9	-		**<0.001**	-		0.663
ICU stay	103	(20.9)	324	(16.2)	26	(20.8)	**1.4**	**(1.07–1.76)**	**0.013**	1.0	(0.62–1.64)	1.000
ICU LOS (days)	9.2	±13.1	8.1	±11.3	9.4	±11.3	-		0.430	-		0.941

AIS = Abbreviated Injury Scale; AOR = adjusted odds ratio; CO = Co-morbidities; CAD = coronary artery disease; CHF = congestive heart failure; CI = confidence interval; CVA = cerebral vascular accident; DM = diabetes mellitus; ESRD = end-stage renal disease; GCS = Glasgow Coma Scale; HTN = hypertension; ICU = intensive care unit; ISS = injury severity score; LOS = length of stay; OR = odds ratio; SD = standard deviation; y/o = years old. One datum of the serum hemoglobin level was missing in the elderly patients without hyponatremia. The values with significant difference between groups are expressed in bold.

**Table 2:** Physiological response and procedures performed upon arrival at the emergency department.

Variable (Fall < 1 m)	Patients ≥ 65 y/o with Hyponatremia n = 492		Patients ≥ 65 y/o without Hyponatremia n = 2002		Patients 20–64 y/o with Hyponatremia n = 125		OR (95% CI) *p* Patients ≥ 65 y/o with Hyponatremia vs. Patients ≥ 65 y/o without Hyponatremia			OR (95% CI) *p* Patients ≥ 65 y/o with Hyponatremia vs. Patients 20–64 y/o with Hyponatremia		
Physiology at ED, n (%)												
GCS < 13	37	(7.5)	121	(6.0)	15	(12.0)	1.3	(0.86–1.85)	0.255	0.6	(0.32–1.13)	0.147
SBP < 90 mmHg	9	(1.8)	18	(0.9)	34	(27.2)	2.1	(0.92–4.60)	0.087	**0.1**	**(0.02–0.11)**	**<0.001**
Heart rate > 100 beats/min	86	(17.5)	324	(16.2)	2	(1.6)	1.1	(0.85–1.43)	0.497	**13.0**	**(3.16–53.70)**	**<0.001**
Procedures at ED, n (%)												
Intubation	12	(2.4)	21	(1.0)	4	(3.2)	**2.4**	**(1.15–4.83)**	**0.025**	0.8	(0.24–2.39)	0.752
Chest tube insertion	1	(0.2)	7	(0.3)	0	(0.0)	0.6	(0.07–4.73)	0.708	-		1.000
Blood transfusion	18	(3.7)	48	(2.4)	3	(2.4)	1.5	(0.89–2.68)	0.156	1.5	(0.45–5.33)	0.593

ED = emergency department; GCS = Glasgow Coma Scale; SBP = systolic blood pressure. The values with significant difference between groups are expressed in bold.

**Table 3:** Significantly associated injuries among elderly patients with hyponatremia.

Variables (Fall < 1 m)	Patients ≥ 65 y/o with Hyponatremia n = 492		Patients ≥ 65 y/o without Hyponatremia n = 2002		Patients 20–64 y/o with Hyponatremia n = 125		OR (95% CI) *p* Patients ≥ 65 y/o with Hyponatremia vs. Patients ≥ 65 y/o without Hyponatremia			OR (95% CI) *p* Patients ≥ 65 y/o with Hyponatremia vs. Patients 20–64 y/o with Hyponatremia		
Cranial fracture	6	(1.2)	38	(1.9)	7	(5.6)	0.6	(0.27–1.52)	0.347	**0.2**	**(0.07–0.63)**	**0.007**
Epidural hematoma (EDH)	7	(1.4)	27	(1.3)	7	(5.6)	1.1	(0.46–2.44)	1.000	**0.2**	**(0.08–0.71)**	**0.012**
Intracerebral hematoma (ICH)	8	(1.6)	34	(1.7)	6	(4.8)	1.0	(0.44–2.08)	1.000	**0.3**	**(0.11–0.96)**	**0.044**
Thoracic vertebral fracture	9	(1.8)	15	(0.7)	0	(0.0)	**2.5**	**(1.07–5.67)**	**0.038**	-	0.216	
Radial fracture	23	(4.7)	222	(11.1)	4	(3.2)	**0.4**	**(0.25–0.61)**	**<0.001**	1.5	(0.50–4.37)	0.627
Ulnar fracture	10	(2.0)	91	(4.5)	4	(3.2)	**0.4**	**(0.23–0.84)**	**0.015**	0.6	(0.19–2.04)	0.498
Femoral fracture	280	(56.9)	1070	(53.4)	33	(26.4)	1.2	(0.94–1.40)	0.173	**3.7**	**(2.38–5.69)**	**<0.001**

The values with significant difference between groups are expressed in bold.

## Supplementary Materials

The following are available online at  www.mdpi.com/1660-4601/14/5/460/s1, Table S1: All injuries among elderly patients with hyponatremia.
