# Supplementary material for: Hyponatremia Is Associated with Worse Outcomes from Fall Injuries in the Elderly
Source: Int J Environ Res Public Health. 2017 Apr 26;14(5):460. doi: 10.3390/ijerph14050460 (PMC5451911; doi:10.3390/ijerph14050460)
Supplement: Supplementary file 1 [file ijerph-14-00460-s001.pdf]

Hyponatremia is Associated with Worse Outcomes from Fall Injuries in the Elderly

Spencer C.H. Kuo, Pao-Jen Kuo, Cheng-Shyuan Rau, Shao-Chun Wu, Shiun-Yuan Hsu and Ching-Hua Hsieh

Supplementary Materials

Table S1. All injuries among elderly patients with hyponatremia.

**Variables (Fall<1M)**	**Patients ≥ 65 y/o with hyponatremia n = 492**	**Patients ≥ 65 y/o without hyponatremia n = 2,002**	**Patients 20–64 y/o with hyponatremia n = 125**	***OR (95%CI)*** **Patients ≥ 65 y/o with hyponatremia vs. Patients ≥ 65 y/o without hyponatremia**	***p***	***OR (95%CI)*** **Patients ≥ 65 y/o with hyponatremia vs. Patients 20–64 y/o with hyponatremia**	***p***
Head trauma, n (%)							
Neurologic deficit	5 (1.0)	9 (0.4)	0 (0.0)	2.3 (0.76–6.81)	0.169	–	0.380
Cranial fracture	6 (1.2)	38 (1.9)	7 (5.6)	0.6 (0.27–1.52)	0.347	**0.2 (0.07–0.63)**	**0.007**
Epidural hematoma (EDH)	7 (1.4)	27 (1.3)	7 (5.6)	1.1 (0.46–2.44)	1.000	**0.2 (0.08–0.71)**	**0.012**
Subdural hematoma (SDH)	60 (12.2)	206 (10.3)	22 (17.6)	1.2 (0.89–1.65)	0.222	0.7 (0.38–1.11)	0.139
Subarachnoid hemorrhage (SAH)	27 (5.5)	95 (4.7)	11 (8.8)	1.2 (0.75–1.81)	0.560	0.6 (0.29–1.25)	0.209
Intracerebral hematoma (ICH)	8 (1.6)	34 (1.7)	6 (4.8)	1.0 (0.44–2.08)	1.000	**0.3 (0.11–0.96)**	**0.044**
Cerebral contusion	27 (5.5)	81 (4.0)	6 (4.8)	1.4 (0.88–2.15)	0.173	1.2 (0.47–2.85)	0.829
Cervical vertebral fracture	0 (0.0)	7 (0.3)	1 (0.8)	–	0.357	–	0.203
Maxillofacial trauma, n (%)							
Orbital fracture	0 (0.0)	2 (0.1)	1 (0.8)	–	1.000	–	0.203
Nasal fracture	0 (0.0)	4 (0.2)	0 (0.0)	–	0.591	–	–
Maxillary fracture	0 (0.0)	11 (0.5)	1 (0.8)	–	0.136	–	0.203
Mandibular fracture	1 (0.2)	3 (0.1)	0 (0.0)	1.4 (0.14–13.08)	1.000	–	1.000
Thoracic trauma, n (%)							
Rib fracture	6 (1.2)	31 (1.5)	4 (3.2)	0.8 (0.33–1.89)	0.682	0.4 (0.10–1.34)	0.226
Sternal fracture	0 (0.0)	0 (0.0)	0 (0.0)	–	–	–	–
Hemothorax	1 (0.2)	9 (0.4)	0 (0.0)	0.5 (0.06–3.57)	0.697	–	1.000

Pneumothorax	1 (0.2)	5 (0.2)	2 (1.6)	0.8 (0.10–6.98)	1.000	0.1 (0.01–1.39)	0.106
Hemopneumothorax	0 (0.0)	3 (0.1)	0 (0.0)	—	0.618	—	—
Lung contusion	0 (0.0)	2 (0.1)	0 (0.0)	—	1.000	—	—
Thoracic vertebral fracture	9 (1.8)	15 (0.7)	0 (0.0)	**2.5 (1.07–5.67)**	**0.038**	—	0.216
Abdominal trauma, n (%)							
Intra-abdominal injury	0 (0.0)	0 (0.0)	0 (0.0)	—	—	—	—
Hepatic injury	0 (0.0)	0 (0.0)	0 (0.0)	—	—	—	—
Splenic injury	1 (0.2)	1 (0.0)	0 (0.0)	4.1 (0.25–65.27)	0.356	—	1.000
Retroperitoneal injury	0 (0.0)	1 (0.0)	0 (0.0)	—	1.000	—	—
Renal injury	0 (0.0)	1 (0.0)	0 (0.0)	—	1.000	—	—
Urinary bladder injury	0 (0.0)	1 (0.0)	0 (0.0)	—	1.000	—	—
Lumbar vertebral fracture	8 (1.6)	33 (1.6)	1 (0.8)	1.0 (0.45–2.15)	1.000	2.1 (0.25–16.54)	0.695
Sacral vertebral fracture	2 (0.4)	1 (0.0)	0 (0.0)	8.2 (0.74–90.25)	0.101	—	1.000
Extremity trauma, n (%)							
Scapular fracture	1 (0.2)	1 (0.0)	0 (0.0)	4.1 (0.25–65.27)	0.356	—	1.000
Clavicle fracture	3 (0.6)	12 (0.6)	1 (0.8)	1.0 (0.29–3.62)	1.000	0.8 (0.08–7.38)	1.000
Humeral fracture	18 (3.7)	103 (5.1)	8 (6.4)	0.7 (0.42–1.17)	0.198	0.6 (0.24–1.31)	0.209
Radial fracture	23 (4.7)	222 (11.1)	4 (3.2)	**0.4 (0.25–0.61)**	**<0.001**	1.5 (0.50–4.37)	0.627
Ulnar fracture	10 (2.0)	91 (4.5)	4 (3.2)	**0.4 (0.23–0.84)**	**0.015**	0.6 (0.19–2.04)	0.498
Metacarpal fracture	0 (0.0)	6 (0.3)	3 (2.4)	—	0.362	—	0.008
Pelvic fracture	5 (1.0)	10 (0.5)	2 (1.6)	2.0 (0.70–6.01)	0.193	0.6 (0.12–3.29)	0.634
Femoral fracture	280 (56.9)	1070 (53.4)	33 (26.4)	1.2 (0.94–1.40)	0.173	**3.7 (2.38–5.69)**	**<0.001**
Tibia fracture	7 (1.4)	33 (1.6)	4 (3.2)	0.9 (0.38–1.96)	0.843	0.4 (0.13–1.52)	0.246
Fibular fracture	2 (0.4)	18 (0.9)	1 (0.8)	0.5 (0.10–1.95)	0.400	0.5 (0.05–5.63)	1.000

The values with significant difference between groups are expressed in bold.
